# p300-Catalyzed Lysine Crotonylation Promotes the Proliferation, Invasion, and Migration of HeLa Cells via Heterogeneous Nuclear Ribonucleoprotein A1

**DOI:** 10.1155/2020/5632342

**Published:** 2020-12-07

**Authors:** Xuesong Han, Xudong Xiang, Hongying Yang, Hongping Zhang, Shuang Liang, Jie Wei, Jing Yu

**Affiliations:** ^1^The First Affiliated Hospital of Kunming Medical University, Kunming, China; ^2^The Third Affiliated Hospital of Kunming Medical University, Kunming, China; ^3^Yunnan Cancer Center, Kunming, China; ^4^Pu'er People's Hospital, Pu'er, China

## Abstract

Cervical carcinoma is the third most common cause of cancer in women with a significant challenge in clinical treatment. Human papillomavirus (HPV) is strongly responsible for cervical carcinoma. Here, we show the increased expression level of heterogeneous nuclear ribonucleoprotein A1 (HNRNPA1) in HPV-associated cervical cancer cells including HeLa, Caski, and SiHa cells, especially in HeLa cells. We provide the evidence that the expression of HNRNPA1 is closely related to HeLa cell proliferation, invasion, and migration. Emerging evidence show that histone modifications account for gene expression. Moreover, our results indicate that HNRNPA1 could be regulated by p300 through p300-mediated lysine crotonylation. Inhibition of p300 downregulated both the lysine crotonylation level and the HNRNPA1 expression. And p300-mediated lysine crotonylation participates in the regulation of HNRNPA1 on HeLa cell proliferation, invasion, and migration. Collectively, our study uncovers that p300-mediated lysine crotonylation enhances expression of HNRNPA1 to promote the proliferation, invasion, and migration of HeLa cells.

## 1. Introduction

Cervical carcinoma is the third most common cause of cancer in women worldwide [1]. Human papillomavirus (HPV) infection is supposed to be a major risk factor of cervical carcinoma [2], and about 95% of cases are caused by infections with high-risk HPV. However, there are limited therapeutic advances for HPV-associated cervical carcinoma. Thus, the prognosis marker of cervical cancers warrants further investigation.

As an oncogene, heterogeneous nuclear ribonucleoprotein A1 (HNRNPA1) was associated with cancer development and was supposed to be a promising therapeutic target in cancer. HNRNPA1 was involved in apoptosis of colon cancer cells [3]. In oral squamous cancer, HNRNPA1 could modulate the cell cycle and proliferation [4]. HNRNPA1 expression was related to the metastasis of breast cancer [5]. And HNRNPA1 activity affected the therapy of hepatocarcinoma [6]. Thus, the function of HNRNPA1 in HPV-associated cervical carcinoma warranted further investigation. Here, we show that HNRNPA1 was upregulated in HPV-associated cervical cells. And inhibition of HNRNPA1 attenuated the proliferation, invasion, and migration of HeLa cells, which indicated that HNRNPA1 represented a potential target for HPV-associated cervical carcinoma.

Proteins such as histones and nonhistones are subject to a vast range of posttranslation modifications (PTMs) which mainly include methylation, phosphorylation, ubiquitination, and acylation. The protein PTMs are identified to play roles in cancer progression such as tumorigenesis [7], oncogenic transformation [8], recurrence [9], and cancer therapy [10]. Moreover, protein PTMs were closely related to gene transcription. Besides acetylation, short-chain Lys acylations have been identified mainly including butyrylation (Kbu), propionylation (Kpr), and crotonylation (Kcr) [11]. More and more evidence demonstrated the role of Kcr in gene transcription [12, 13].

Different protein PTMs were carried out by different enzymes; these enzymes are frequently mutated or dysregulated in various types of cancer [14]. p300, as a transcriptional coactivator, contributed to cell proliferation [15], differentiation [16], apoptosis [17], and autophagy [18]. Except for histone acetyltransferase (HAT) activity of p300 [19], recent studies have illuminated that the histone crotonyltransferase activity of p300 and p300-mediated Kcr directly stimulates transcription [20]. A study has shown that p300 and p300-mediated Kcr participated in HNRNPA1 regulation [21]. However, the role of p300-mediated Kcr in HeLa cell proliferation, invasion, and migration which are regulated by HNRNPA1 remained obscure.

In present study, we investigated the effect of HNRNPA1 on proliferation, invasion, and migration of HeLa cells. Moreover, our results indicated that p300-mediated lysine crotonylation was able to regulate the expression of HNRNPA1 and in turn affect cell development.

## 2. Materials and Methods

### 2.1. Cell Culture

Human cervical cell lines (HeLa cells, Caski cells, and SiHa cells) and the normal cervical epithelial cell line HCerEPiC were purchased from ATCC (American Type Culture Collection, USA). Cells were maintained in Dulbecco's modified Eagle's medium (DMEM) supplemented with 10% fetal bovine serum (FBS) and 1% of penicillin solution. For sodium crotonate (NaCr, Alfa, B22235) treatment, cells were grown to 40% confluency and 20 mM NaCr was added to the medium [22]. Cells were treated with the same concentration of NaCl in parallel as the control.

### 2.2. Cell Transfection

Stable knockdown of target genes was achieved by siRNA. The siRNA sequences used in this study were as follows: sip300 forward: AGAUACAAGCGAGGAAAACCA; reverse: GUUUUCCUCGCUUGUAUCUCC. siACSS2 forward: UUCUUAAAUAUCUAACUCCAA; reverse: GGAGUUAGAUAUUUAAGAAUC. siHNRNPA1 was purchased from Santa Cruz Biotechnology (Santa Cruz, CA). Cells were transfected with siRNA using Lipofectamine 3000 (Invitrogen, # L3000-015) for 24 h according to the manufacturer's protocol. The knockdown efficiency was measured by western blots. For sodium crotonate treatment, transfected cells were grown to 40% confluency and 20 mM NaCr added to medium.

### 2.3. Western Blots

Western blot analysis for a specific protein expression was performed. The protein was separated by sodium dodecyl sulfonate polyacrylamide gel electrophoresis (SDS-PAGE). The protein was transferred to a PVDF membrane by the wet transfer. After being blocked 1 hour in room temperature, the membrane was incubated by primary antibodies incorporating anti-histone H3 (ABclonal, A2348, 1 : 5000), anti-GAPDH (ABclonal, A19056, 1 : 5000), anti-panKcr (PTM-501, 1 : 1000), anti-p300 (Abcam, ab59240, 1 : 2000), anti-ASCC2 (Abcam, ab234689, 1 : 2000), and anti-HNRNPA1 (Abcam, ab4791, 1 : 1000) at room temperature for 2 hours and then incubated with HRP-conjugated anti-rabbit (Abcam, 205717, 1 : 5000) and anti-mouse (Abcam, 205718, 1 : 5000) at room temperature for 1 hour.

### 2.4. Cell Counting Kit (CCK-8) Assay

CCK-8 kit (Solarbio, # CA1210) was used to determine the cell proliferation. HeLa cells (5,000 cells/well) with or without HNRNPA1 knockdown and NaCr treatment were cultured. CCK-8 solution was added, and cell numbers were then counted at 0, 24, 48, 72, and 96 h. Then, the optical density at 450 nm was measured by a microtiter plate reader.

### 2.5. Cell Migration and Invasion Assay

The cell migration and invasion assays were performed with Transwell chambers (Corning). For the migration assay, HeLa cells (2 × 10^4^ in each well) with or without treatments were seeded in the upper Transwell chambers, and the lower chamber was filled with a medium containing 10% FBS. For the invasion assay, the upper compartment was precoated with 100 *μ*l of Matrigel. After 24 h incubation, the cells in the upper surface were removed. Cells in the lower chamber were fixed with methanol and stained with Giemsa (Solarbio, # G4640). The invasion and migration of HeLa cells were quantified using the Image-Pro Plus 6.0.

### 2.6. Statistical Analysis

All quantitative data are presented as the mean ± SEM, and the differences were analyzed by two-tailed Student's *t* test or one-way ANOVA. One-way ANOVA with the Tukey post hoc test was used to analyze the differences among the different groups. Student's *t* test was used for comparisons between two groups. Significance was accepted at *P* < 0.05.

## 3. Results and Discussion

### 3.1. Knockdown of HNRNPA1 Attenuated the Proliferation, Invasion, and Migration of HeLa Cells

**Figure 1:**
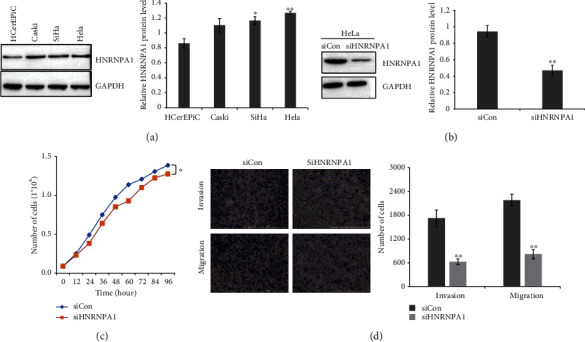
Knockdown of HNRNPA1 attenuated the proliferation, invasion, and migration of HeLa cells. (a) Western blot results showed the expression of HNRNPA1 in normal cervical epithelial cells (HCerEPiC) and HPV-associated cervical cancer cells (HeLa cells, Caski cells, and SiHa cells). The relative intensities of HNRNPA1/GAPDH were quantified using ImageJ. ^∗^*P* < 0.05; ^∗∗^*P* < 0.01. (b) HNRNPA1 expression was measured by western blot in HeLa cells stably expressing nontargeting control (siCon) and siHNRNPA1. The relative intensities of HNRNPA1/GAPDH were quantified using ImageJ. ^∗∗^*P* < 0.01. (c) Effects of HNRNPA1 on HeLa cell proliferation were determined by CCK-8 kits. ^∗^*P* < 0.05. (d) Transwell assay was performed to determine the invasion and migration of HeLa cells. The invasion and migration of HeLa cells were quantified using Image-Pro Plus 6.0. ^∗^*P* < 0.05; ^∗∗^*P* < 0.01.

To identify the HNRNPA1 level in HPV-associated cervical cancer cells, we performed western blot assays to detect the protein level of HNRNPA1 in HeLa cells, Caski cells, SiHa cells, and HCerEPiC cells. As shown in Figure 1(a), the elevated level of HNRNPA1 could be observed in HPV-associated cervical cancer cells, particularly in HeLa cells. In order to determine the role of HNRNPA1 in HeLa cell development, HeLa cells expressing siRNA targeting HNRNPA1 were used (Figure 1(b)). As shown in Figure 1(c), inhibition of HNRNPA1 attenuated HeLa cell proliferation. In addition, HNRNPA1 silencing suppressed invasion and migration of HeLa cells (Figure 1(d)). Thus, our studies indicated that HNRNPA1 was involved in HeLa cell progression.

### 3.2. p300-Mediated Lysine Crotonylation in HeLa Cells

**Figure 2:**
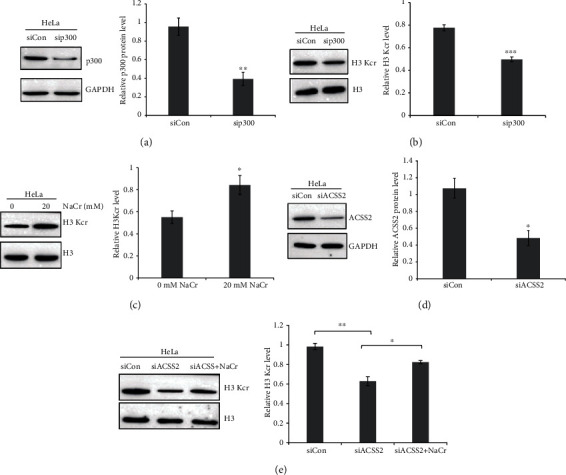
p300-mediated lysine crotonylation in HeLa cells. (a) p300 expression was measured by western blot in HeLa cells stably expressing nontargeting control (siCon) and sip300. The relative intensities of p300/GAPDH were quantified using ImageJ. ^∗∗^*P* < 0.01. (b) H3 Kcr expression was measured by western blot in HeLa cells stably expressing nontargeting control (siCon) and sip300. The relative intensities of H3 Kcr/H3 were quantified using ImageJ. ^∗∗∗^*P* < 0.001. (c) Effect of NaCr on H3 Kcr expression was determined by western blot. The relative intensities of H3 Kcr/H3 were quantified using ImageJ. ^∗^*P* < 0.05. (d) ACSS2 expression was measured by western blot in HeLa cells stably expressing nontargeting control (siCon) and siACSS2. The relative intensities of ACSS2/GAPDH were quantified using ImageJ. ^∗∗^*P* < 0.01. (e) H3 Kcr expression was determined by western blot in HeLa cell-treated siACSS2 or/and NaCr. The relative intensities of H3 Kcr/H3 were quantified using ImageJ. ^∗^*P* < 0.05; ^∗∗^*P* < 0.01.

In order to determine whether p300 is responsible for Kcr in HeLa cells as reported, HeLa cells expressing siRNA targeting p300 were used. The knockdown efficiency of p300 was validated by western blot (Figure 2(a)). And p300 deficiency reduced the Kcr level in HeLa cells (Figure 2(b)). Based on the former result, p300-mediated Kcr could be induced by additional crotonyl-CoA, which was generated from NaCr [20]. When HeLa cells were treated with different concentrations of NaCr, the increased level of Kcr could be observed (Figure 2(c)). Oppositely, knockdown acyl-CoA synthetase (ACSS2), which was known to produce acetyl-CoA, resulted in reduced Kcr level (Figures 2(d) and 2(e)). And NaCr administration in ACSS2-deficient HeLa cells could rescue the Kcr level (Figure 2(e)).

### 3.3. p300 and Lysine Crotonylation Is Responsible for HNRNPA1 Expression in HeLa Cells

**Figure 3:**
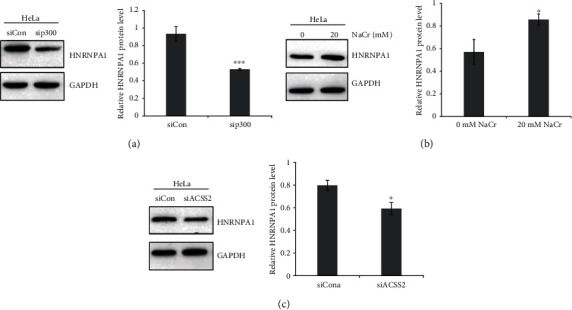
p300-mediated lysine crotonylation is responsible for HNRNPA1 expression in HeLa cells. (a) HNRNPA1 expression was measured by western blot in HeLa cells stably expressing nontargeting control (siCon) and sip300. The relative intensities of HNRNPA1/GAPDH were quantified using ImageJ. ^∗∗∗^*P* < 0.001. (b) HNRNPA1 expression was measured by western blot in NaCr-treated HeLa cells. The relative intensities of HNRNPA1/GAPDH were quantified using ImageJ. ^∗^*P* < 0.05. (c) HNRNPA1 expression was measured by western blot in HeLa cells stably expressing nontargeting control (siCon) and siACSS2. The relative intensities of HNRNPA1/GAPDH were quantified using ImageJ. ^∗^*P* < 0.05.

Accumulating evidence indicated the effect of Kcr on gene transcription [23, 24]. It has been demonstrated that HNRNPA1 was a p300-regulated lysine crotonylation protein [21]. In order to validate whether the expression of HNRNPA1 could be affected by Kcr, we performed a western blot assay to detect the HNRNPA1 protein level under p300 knockdown. The expression level of HNRNPA1 was significantly decreased in HeLa cell transfected sip300 (Figure 3(a)). In addition, the expression level of HNRNPA1 could be upregulated by NaCr treatment (Figure 3(b)). And inhibition of ACSS2 led to decreased HNRNPA1 expression level (Figure 3(c)). In a word, our studies suggested that p300-catalyzed Kcr was involved in regulation of HNRNPA1 expression.

### 3.4. p300-Mediated Lysine Crotonylation Is Involved in the Regulation of HNRNPA1 on Proliferation, Invasion, and Migration of HeLa Cells

**Figure 4:**
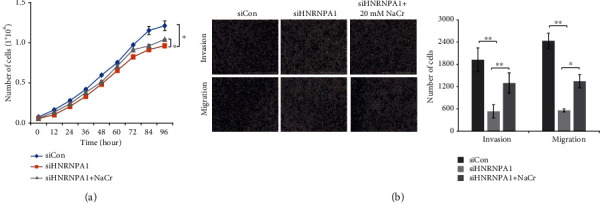
p300-mediated lysine crotonylation is involved in the regulation of HNRNPA1 on proliferation, invasion, and migration of HeLa cells. (a) Effects of HNRNPA1 or/and NaCr on HeLa cell proliferation were determined by CCK-8 kit. ^∗^*P* < 0.05. (b) Transwell assay was performed to determine the invasion and migration of HeLa cells treated with siHNRNPA1 or/and NaCr. The invasion and migration of HeLa cells were quantified using Image-Pro Plus 6.0. ^∗^*P* < 0.05; ^∗∗^*P* < 0.01; ^∗∗∗^*P* < 0.001.

In order to determine the role of p300-catalyzed Kcr on the regulation of HNRNPA1 on HeLa cell progression, NaCr was used to treat HeLa cells with siHNRNPA1. As shown in Figure 4(a), NaCr administration rescued the inhibitory effect of HNRNPA1 knockdown on cell proliferation. Moreover, NaCr treatment also recovered the invasion and migratory abilities of HeLa cells with siHNRNPA1 (Figures 4(b) and 4(c)).

## 4. Conclusions

In the present work, we have demonstrated the function of HNRNPA1 in HPV-associated cervical cancer. The expression of HNRNPA1 was responsible for the development of HeLa cells. Knockdown of HNRNPA1 attenuated cell proliferation, invasion, and migration. We then detect the effect of p300 and p300-mediated Kcr on HNRNPA1 and Hele cell development. Inhibition of p300 dampened the Kcr level and HNRNPA1 expression. However, as we can see from Figure 2(b), knockdown of p300 could not completely abolish the histone H3 Kcr level, which indicated that the involvement of CBP on Kcr. NaCr administration was able to induce Kcr and promote HNRNPA1 expression. On the contrary, the inhibition of crotonyl-CoA provider ACSS2 led to Kcr and HNRNPA1 reduction. Thus, our results indicated that p300-mediated Kcr was involved in HNRNPA1 expression. In order to identify the role of p300-mediated Kcr in HNRNPA1-regulated HeLa cell development, we performed proliferation assay and migration assay under NaCr administration. NaCr treatment attenuated the inhibitory effect of HNRNPA1 knockdown in HeLa cell proliferation, invasion, and migration.

Taken together, our investigation demonstrated that HNRNPA1 enhanced cell proliferation, invasion, and migration of HeLa cells through p300-mediated lysine crotonylation.

## Data Availability

No data were used to support this study.
